# Association of plasma aldosterone concentration with peripheral artery disease in hypertensive patients: insights from a large cross-sectional analysis

**DOI:** 10.3389/fcvm.2025.1549878

**Published:** 2025-03-25

**Authors:** Yingying Zhang, Xintian Cai, Shuaiwei Song, Junli Hu, Pan Zhou, Kangxin Cai, Rui Ma, Huimin Ma, Di Shen, Wenbo Yang, Delian Zhang, Qin Luo, Jing Hong, Nanfang Li

**Affiliations:** ^1^ Hypertension Center of People’s Hospital of Xinjiang Uygur Autonomous Region, Urumqi, Xinjiang, China; ^2^ Xinjiang Hypertension Institute, Urumqi, Xinjiang, China; ^3^ NHC Key Laboratory of Hypertension Clinical Research, Urumqi, Xinjiang, China; ^4^ Key Laboratory of Xinjiang Uygur Autonomous Region ”Hypertension Research Laboratory”, Urumqi, Xinjiang, China; ^5^ Hypertension Research Laboratory, Urumqi, Xinjiang, China; ^6^ Xinjiang Clinical Medical Research Center for Hypertension (Cardio-Cerebrovascular) Diseases, Urumqi, Xinjiang, China

**Keywords:** plasma aldosterone concentration, peripheral vascular disease, ankle-brachial index, hypertension, non-linear relationship, cross-sectional study

## Abstract

**Objectives:**

To investigate the relationship between plasma aldosterone concentration (PAC) and the prevalence of peripheral artery disease (PAD) in hypertensive patients and to determine any potential threshold effects.

**Methods:**

This cross-sectional study analyzed data from 13,157 hypertensive individuals from the People's Hospital of Xinjiang Uygur Autonomous Region, China. PAD was diagnosed based on an ankle-brachial index (ABI) of ≤0.90. A multivariate logistic regression model was utilized to evaluate the association between PAC and PAD, and a generalized additive model (GAM) was employed to explore non-linear relationships.

**Results:**

The fully adjusted logistic regression model revealed a significant positive association between PAC and PAD, with an odds ratio (OR) [95% confidence interval (CI)] of 1.06 (1.04, 1.08) per unit increase in PAC. The GAM identified a critical threshold at 17.00 ng/dl for PAC, above which the prevalence of PAD increased by 9% for each unit increase in PAC, with an OR (95% CI) of 1.09 (1.06, 1.11). Sensitivity and subgroup analyses confirmed the robustness of these findings.

**Conclusion:**

This study establishes a non-linear relationship between PAC and the prevalence of PAD in hypertensive patients, with a critical threshold at 17.00 ng/dl. These findings underscore the importance of aldosterone homeostasis in vascular health and the need for further large-scale, prospective studies to validate these results and explore their clinical implications.

## Introduction

Ranking as the third most prevalent atherosclerotic condition globally, peripheral artery disease (PAD) is surpassed only by coronary heart disease (CHD) and stroke in terms of incidence (1). The global burden of PAD is substantial, with an estimated 202 million individuals affected, highlighting its emergence as a significant public health challenge with considerable economic implications (2, 3). PAD not only imposes limb-threatening complications, such as intermittent claudication, ischemic rest pain, ulceration, gangrene, and significant functional decline, but also leads to a significant increase in cardiovascular morbidity and mortality (4–6). Hypertension, a preventable risk factor, plays a crucial role in the development of PAD (1, 7–9). A nationally representative cross-sectional health survey has indicated that the prevalence of PAD is disproportionately higher by 68% in untreated hypertensive individuals compared to the general population, with an odds ratio (OR) and 95% confidence intervals (CIs) of 1.68 (1.13, 2.50). However, for people with treated but uncontrolled hypertension, the prevalence of PAD was 95% higher than for the general population, with an OR of 1.95 (1.40, 2.72) (10). Furthermore, the research by Korhonen et al. has underscored the particular vulnerability of individuals with resistant hypertension to PAD, exhibiting a higher risk than those with well-managed hypertension (11). Therefore, identifying and mitigating potential risk factors for PAD in hypertensive individuals is critical to prevent adverse cardiovascular outcomes and limb-related sequelae.

Aldosterone, a mineralocorticoid hormone produced by the zona glomerulosa of the adrenal cortex, is crucial for the regulation of arterial blood pressure and maintenance of fluid and electrolyte balance (12). Primary aldosteronism (PA) is characterized by the autonomous and excessive secretion of aldosterone by the adrenal cortex (13). This hormonal imbalance is associated with more severe arterial wall damage compared to essential hypertension (14, 15). Numerous studies has consistently demonstrated that aldosterone promotes the local production of vasoconstrictive agents, including endothelin (ET), and angiotensin II (Ang II), thereby contributing to endothelial dysfunction (16). There is a significant correlation between elevated plasma aldosterone concentrations (PAC) and the presence of subclinical atherosclerosis, inflammation, oxidative stress, and endothelial dysfunction (17, 18). As a recognized fundamental pathophysiological mechanism of vascular disease, atherosclerosis is also a major pathological manifestation of PAD (19). These associations suggest that elevated PAC may actively promote atherosclerosis, inflammation, oxidative stress, and endothelial dysfunction, thereby contributing to the development or progression of PAD (20, 21).

We hypothesize that aldosterone may serve as a modifiable risk factor for PAD, particularly among hypertensive patients. To explore this hypothesis, we conducted a cross-sectional study to investigate the correlation between PAC and the prevalence of PAD within this specific demographic. Our findings could potentially identify a novel therapeutic target for the intervention of PAD in hypertensive individuals.

## Material and methods

### Study design and participant selection

**Figure 1:**
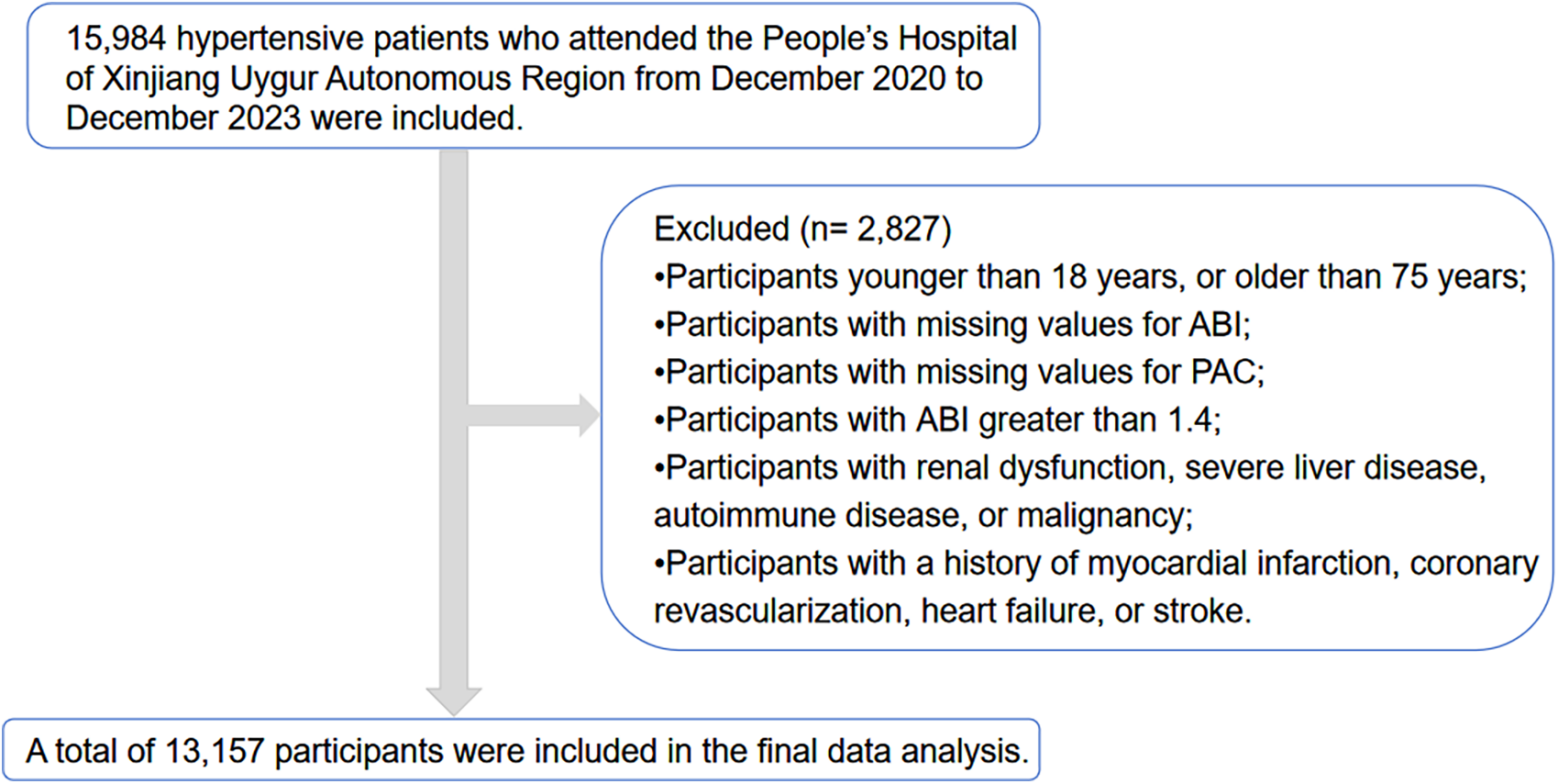
Flow chart of study participants.

The present cross-sectional study leveraged data extracted from the electronic medical records at the People's Hospital of Xinjiang Uygur Autonomous Region, China, conducted over a three-year interval from December 2020 to December 2023. The study encompassed an initial pool of 15,984 subjects, from which of 13,157 hypertensive patients was selected for the final analysis, as depicted in Figure 1. Eligibility was determined for individuals (18-to-75 years old) with a diagnosis of hypertension. Exclusion criteria were as follows: 1. Participants with incomplete data for ankle-brachial index (ABI) and PAC; 2. Individuals with an ABI greater than 1.4 were excluded due to the potential for arterial wall calcification, which can falsely elevate ABI values (22, 23); 3. Participants with renal impairment, severe hepatic disease, autoimmune disorders, or malignancy; 4. Individuals with a history of myocardial infarction, coronary revascularization, heart failure, or stroke. This study, conducted in alignment with the ethical principles of the Declaration of Helsinki, received ethical approval from the institutional review board, reference number KY2024072536. Informed consent was secured from all participants prior to their inclusion in the study.

### Data collection and parameter definition

We extracted a comprehensive dataset from the electronic medical records of patients at the People's Hospital of Xinjiang Uygur Autonomous Region, China. The data included demographic data, anthropometric measures, and biochemical profiles. Demographic data comprised age, gender, smoking habits, and alcohol consumption. Anthropometric indices included body mass index (BMI), systolic blood pressure (SBP), and diastolic blood pressure (DBP). Biochemical assays encompassed fasting plasma glucose (FPG), lipid profiles (high-density lipoprotein cholesterol [HDL-C], low-density lipoprotein cholesterol [LDL-C], total cholesterol [TC], and triglycerides [TG]), serum uric acid (SUA), blood urea nitrogen (BUN), serum creatinine (Scr), liver enzymes [aspartate aminotransferase [AST] and alanine aminotransferase [ALT]], serum potassium (K^+^), and homocysteine (Hcy). Comorbidities such as diabetes mellitus, hyperlipidemia, and CHD were recorded, along with medication use, specifically antihypertensives, lipid-lowering agents, antidiabetics, and antiplatelet drugs.

PAC was measured using the DSL-8600 ACTIVE Aldosterone Coated Tube Radioimmunoassay Kit, adhering to clinical guidelines and our prior research (24–30). Plasma renin activity (PRA) was quantified using an iodine angiotensin I radioimmunoassay kit. The aldosterone/renin ratio (ARR) was calculated as the ratio of plasma aldosterone to PRA. Supplementary Material provides further details on data collection protocols and definitions.

### Measurement of ABI and definition of PAD

Participants rested in a controlled-temperature setting for a minimum of 10 min, with limbs exposed to standardize conditions. Consumption of stimulants was prohibited 30 min prior to measurement to minimize vasoactive effects. ABI was measured using the Omron Colin BP-203RPE III device (Omron Health Care, Kyoto, Japan) by trained technicians, who obtained simultaneous SBP readings from the brachial and ankle arteries in the supine position, in accordance with the manufacturer's guidelines (31). ABI was calculated as the ratio of ankle SBP to brachial SBP, with the device's software automatically computing bilateral values for accuracy and reproducibility. The lower ABI value was used for each participant in analysis. PAD was diagnosed if the ABI was ≤0.9 in either lower limb, following standard diagnostic criteria (23, 32).

### Statistical analysis

Participants were stratified into tertiles by PAC levels. Multicollinearity was assessed using variance inflation factors, detailed in Supplementary Table S1. Univariate logistic regression analysis was performed to assess the impact of the clinical and biochemical indicators on the risk of PAD (Supplementary Table S2). We applied multivariate logistic regression to calculate ORs and 95% CIs for the association between PAC tertiles and PAD prevalence. A generalized additive model (GAM) was used to explore nonlinear relationships between PAC and PAD, with a recursive partitioning algorithm identifying inflection points for nonlinearity. Subgroup analyses were conducted to uncover additional risk factors that could modulate the PAC-PAD association. Sensitivity analyses were conducted to ascertain the robustness of our findings. The Supplementary Material includes a full description of the statistical methods. All tests were two-tailed, with significance set at *α* = 0.05, and analyses were conducted using R software (version 4.1.1).

## Results

### Characteristics of participants by PAC tertiles

**Figure 2:**
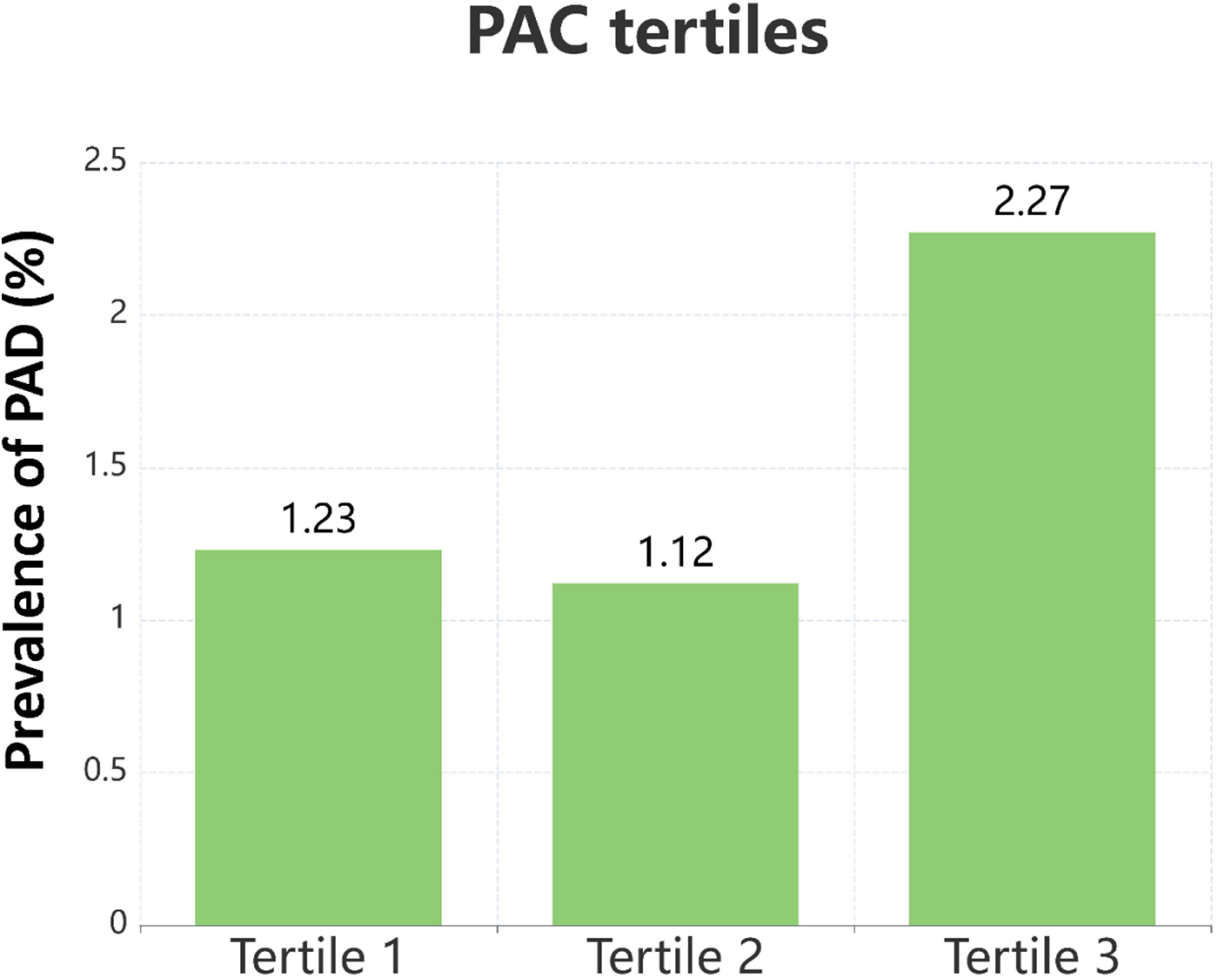
Bar chart represented the prevalence of PAD across different tertiles of PAC.

Table 1 presents the demographic and clinical characteristics of the study population, categorized by PAC tertiles. Participants in the upper PAC tertiles were predominantly female and younger, with higher levels of SBP, DBP, FPG, HDL-C, BUN, Scr, PRA, and ARR. There was also a higher prevalence of calcium channel blocker and diuretic use in these groups. In contrast, a lower proportion of individuals in the upper PAC tertiles were current smokers or drinkers. No statistically significant differences were found across PAC tertiles for BMI, TC, TG, SUA, LDL-C, AST/ALT, Hcy, hyperlipidemia, antiplatelet drugs, lipid-lowering drugs, or comorbidities. The distribution of PAC into tertiles facilitated an analysis of PAD prevalence across these groups, as shown in Figure 2. The prevalence rates were 1.23% for the first tertile (T1), 1.12% for the second tertile (T2), and 2.27% for the third tertile (T3).

**Table 1:** Baseline characteristics of participants according to PAC tertiles.

Tertiles of PAC	Tertile 1	Tertile 2	Tertile 3	*P* value
	(<12.65)	(12.65–17.45)	(>17.45)	
Participants, *n*	4,378	4,379	4,400	
Age, years	53.52 ± 10.73	53.08 ± 10.54	52.53 ± 10.69	<0.001
Female, *n* (%)	1,501 (34.29%)	1,876 (42.84%)	2,021 (45.93%)	<0.001
BMI, kg/m^2^	27.04 ± 3.58	27.07 ± 3.51	27.06 ± 3.62	0.907
SBP, mmHg	146.55 ± 18.60	147.04 ± 18.46	148.78 ± 19.52	<0.001
DBP, mmHg	89.04 ± 13.15	90.39 ± 13.13	91.27 ± 13.82	<0.001
Current smoking, *n* (%)	1,852 (42.30%)	1,476 (33.71%)	1,301 (29.57%)	<0.001
Current drinking, *n* (%)	1,610 (36.77%)	1,333 (30.44%)	1,259 (28.61%)	<0.001
Laboratory results				
FPG, mmol/L	5.09 ± 0.98	5.12 ± 0.95	5.18 ± 1.00	<0.001
HDL-C, mmol/L	1.08 (0.92–1.28)	1.09 (0.93–1.30)	1.10 (0.94–1.30)	0.002
LDL-C, mmol/L	2.83 (2.22–3.41)	2.83 (2.23–3.46)	2.85 (2.23–3.44)	0.643
TC, mmol/L	4.58 (3.88–5.29)	4.60 (3.92–5.35)	4.60 (3.91–5.33)	0.190
TG, mmol/L	1.55 (1.09–2.29)	1.55 (1.10–2.29)	1.57 (1.11–2.23)	0.324
SUA, umol/L	358.07 ± 94.58	356.72 ± 93.43	359.98 ± 92.38	0.258
BUN, mmol/L	5.09 ± 1.37	5.13 ± 1.39	5.20 ± 1.48	<0.001
Scr, umol/L	63.74 ± 14.29	64.09 ± 15.02	65.82 ± 16.38	<0.001
AST/ALT	0.89 ± 0.32	0.88 ± 0.31	0.88 ± 0.31	0.142
Hcy, mmol/L	14.32 ± 4.84	14.11 ± 4.63	14.30 ± 4.69	0.065
K^+^, mmol/L	3.91 ± 0.33	3.87 ± 0.32	3.82 ± 0.35	<0.001
PRA, ng/ml/h	1.87 (0.85–2.96)	2.06 (1.00–3.35)	2.60 (1.48–4.25)	<0.001
PAC, ng/dl	10.84 (9.91–11.71)	14.77 (13.66–16.00)	21.56 (19.15–25.90)	<0.001
ARR	5.74 (3.54–12.62)	7.21 (4.43–14.81)	8.56 (5.29–15.83)	<0.001
Comorbidities, *n* (%)				
Diabetes	731 (16.70%)	706 (16.12%)	787 (17.89%)	0.080
CHD	391 (8.93%)	344 (7.86%)	368 (8.36%)	0.192
Hyperlipidemia	2,273 (51.92%)	2,311 (52.77%)	2,292 (52.09%)	0.698
Medication use, *n* (%)				
ACEIs/ARBs	2,235 (51.05%)	2,111 (48.21%)	2,186 (49.68%)	0.029
Beta-blockers	943 (21.54%)	814 (18.59%)	934 (21.23%)	<0.001
Calcium channel blockers	2,624 (59.94%)	2,756 (62.94%)	3,174 (72.14%)	<0.001
Diuretics	674 (15.40%)	782 (17.86%)	1,260 (28.64%)	<0.001
Lipid-lowering drugs	845 (19.30%)	786 (17.95%)	874 (19.86%)	0.064
Antidiabetic drugs	423 (9.66%)	339 (7.74%)	433 (9.84%)	<0.001
Antiplatelet drugs	650 (14.85%)	600 (13.70%)	649 (14.75%)	0.239

Data are mean ± SD, median (IQR) for skewed variables or numbers (proportions) for categorical variables.

BMI, body mass index; SBP, systolic blood pressure; DBP, diastolic blood pressure; FPG, fasting plasma glucose; HDL-C, high-density lipoprotein cholesterol; LDL-C, low-density lipoprotein cholesterol; TC, total cholesterol; TG, total triglyceride; SUA, serum uric acid; BUN, blood urea nitrogen; Scr, serum creatinine; AST, aspartate aminotransferase; ALT, alanine aminotransferase; Hcy, homocysteine; K^+^, serum potassium; PRA, plasma renin activity; ARR, aldosterone/renin ratio; CHD, coronary heart disease; ACEIs, angiotensin-converting enzyme inhibitors; ARBs, angiotensin receptor blockers, PAD, peripheral artery disease.

### Association between PAC and the prevalence of PAD

We constructed four logistic regression models to assess the correlation between PAC and PAD (Table 2). The crude model revealed an OR of 1.07 (1.05, 1.08), suggesting a 7% increase in PAD prevalence for each unit increase in PAC. After full adjustment, model 3 indicated a 6% increase in PAD prevalence per unit increase in PAC. Table 2 illustrates the positive association between PAC and PAD prevalence, which attenuated but remained significant with progressive adjustment. To conduct a trend analysis, PAC was categorized into tertiles. The ORs (95% CI) for the T2 and T3 compared with the T1 were 0.91 (0.62, 1.35) and 1.68 (1.18, 2.38), respectively. There was a consistent, statistically significant increase in PAD prevalence across PAC tertiles, with the highest tertile showing the greatest OR compared to the lowest (*P* for trend <0.05).

**Table 2:** Association of PAC with the prevalence of PAD.

Exposure	Crude Model		Model 1		Model 2		Model 3	
	OR (95% CI)	*P* value	OR (95% CI)	*P* value	OR (95% CI)	*P* value	OR (95% CI)	*P* value
PAC (Per unit increase)	1.07 (1.05, 1.08)	<0.001	1.07 (1.05, 1.08)	<0.001	1.07 (1.05, 1.08)	<0.001	1.06 (1.04, 1.08)	<0.001
Tertiles of PAC								
Tertile 1	Reference		Reference		Reference		Reference	
Tertile 2	0.91 (0.61, 1.34)	0.620	0.91 (0.62, 1.35)	0.649	0.91 (0.61, 1.35)	0.639	0.91 (0.62, 1.35)	0.649
Tertile 3	1.86 (1.33, 2.60)	0.001	1.89 (1.35, 2.64)	0.001	1.77 (1.25, 2.49)	0.001	1.68 (1.18, 2.38)	0.004
*P* for trend	<0.001		<0.001		0.001		0.002	

Crude Model: Unadjusted; Model 1: Adjust for age, sex, current smoking, current drinking, diabetes, CHD, hyperlipidemia, BMI, SBP, DBP; Model 2: Model 1 plus adjustment for FPG, HDL-C, LDL-C, TC, TG, SUA, BUN, Scr, AST/ALT, Hcy, K^+^; Model 3: Model 2 plus adjustment for ACEIs/ARBs, beta blockers, calcium channel blockers, diuretics, lipid-lowering drugs, antidiabetic drugs, antiplatelet drugs.

PAC, plasma aldosterone concentration; PAD, peripheral artery disease; OR, odds ratio; CI, confidence interval.

### Nonlinear and threshold effects of PAC on PAD risk

**Figure 3:**
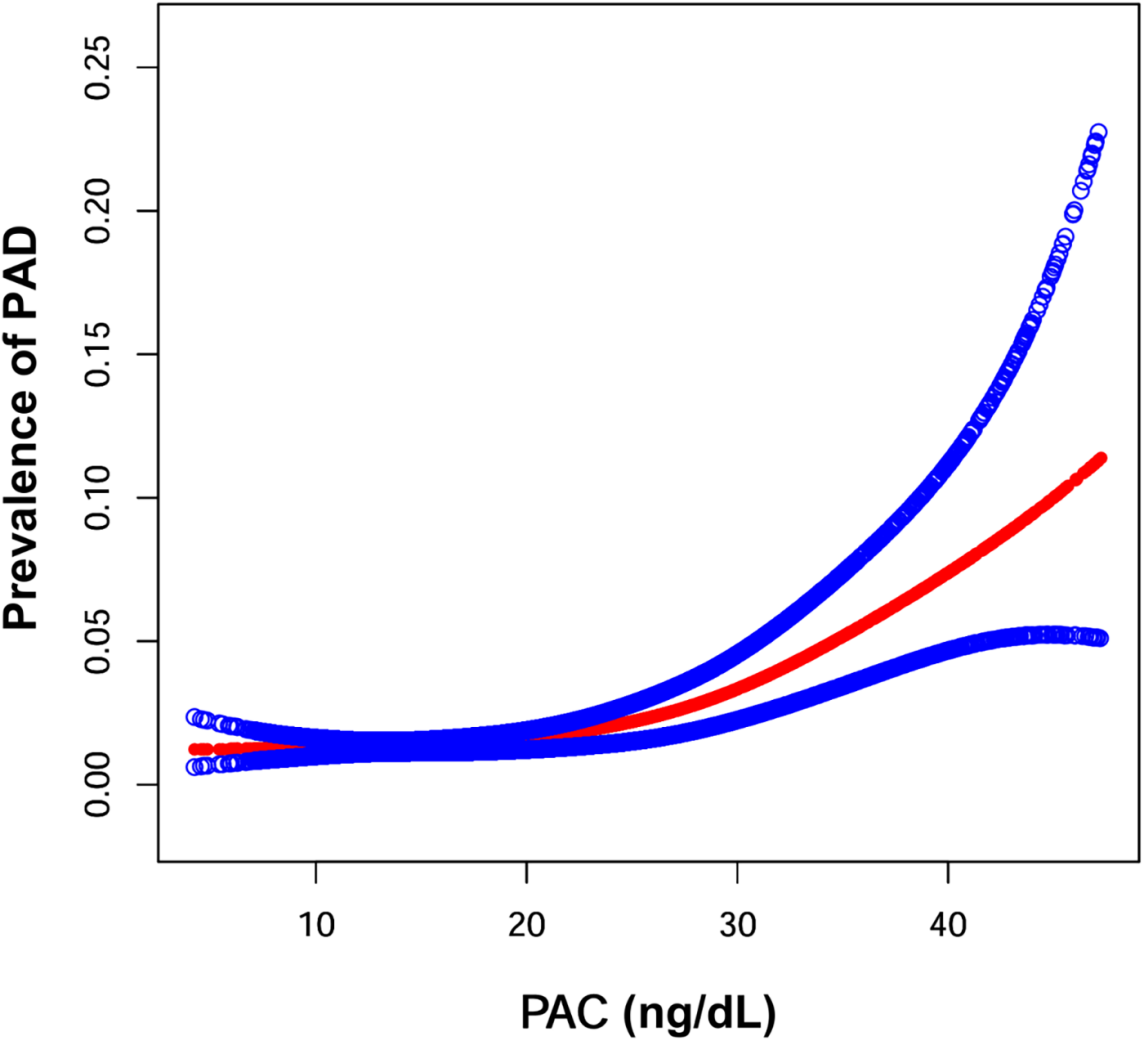
Generalized additive model were performed to examine the shape of the associations between PAC and the prevalence of PAD. Age, sex, smoking state, drinking state, diabetes, coronary heart disease, hyperlipidemia, BMI, SBP, DBP, FPG, HDL-C, LDL-C, TC, TG, SUA, BUN, Scr, AST/ALT, Hcy, K^+^, ACEIs/ARBs, beta blockers, calcium channel blockers, diuretics, lipid-lowering drugs, antidiabetic drugs, antiplatelet drugs were adjusted.

Figure 3 depicts the nonlinear relationship between PAC and PAD prevalence. Utilizing the GAM, we identified a nonlinear correlation and determined the inflection point at 17.00 ng/dl. Above this threshold, there was a 9% increase in PAD prevalence for each unit increase in PAC, with an OR of 1.09 (1.06, 1.11). Below the threshold, the association between PAC and PAD did not reach statistical significance, with an OR of 0.98 (0.92, 1.04) as detailed in Table 3.

**Table 3:** Threshold analyses on PAC and the prevalence of PAD using two-piecewise regression models.

PAC	Crude Model		Model 1		Model 2		Model 3	
	OR (95% CI)	*P* value	OR (95% CI)	*P* value	OR (95% CI)	*P* value	OR (95% CI)	*P* value
<17 (per unit increment)	0.98 (0.92, 1.04)	0.503	0.98 (0.93, 1.04)	0.549	0.98 (0.92, 1.04)	0.461	0.98 (0.92, 1.04)	0.461
≥17 (per unit increment)	1.09 (1.07, 1.11)	<0.001	1.09 (1.07, 1.11)	<0.001	1.09 (1.07, 1.11)	<0.001	1.09 (1.06, 1.11)	<0.001
*P* value for Log-likelihood ratio test	0.004		0.005		0.004		0.006	

Crude Model: Unadjusted; Model 1: Adjust for age, sex, current smoking, current drinking, diabetes, CHD, hyperlipidemia, BMI, SBP, DBP; Model 2: Model 1 plus adjustment for FPG, HDL-C, LDL-C, TC, TG, SUA, BUN, Scr, AST/ALT, Hcy, K^+^; Model 3: Model 2 plus adjustment for ACEIs/ARBs, beta blockers, calcium channel blockers, diuretics, lipid-lowering drugs, antidiabetic drugs, antiplatelet drugs.

PAC, plasma aldosterone concentration; PAD, peripheral artery disease; OR, odds ratio; CI, confidence interval.

### Sensitivity and subgroup analysis

Sensitivity analyses were performed by excluding individuals suspected of having PA, and the stability of our findings was confirmed (Supplementary Table S3). To address the potential impact of outliers, we removed data points for PAC values outside the 1st and 99th percentiles, and the results remained consistent (Supplementary Table S4). To further ensure the robustness of our statistical findings, we excluded participants with incomplete covariate data, and the results were found to be consistent (Supplementary Table S5).

To assess the association between PAC and PAD across diverse populations, we conducted subgroup analyses stratified by age, sex, smoking status, drinking status, hyperlipidemia, diabetes, CHD, BMI, and PRA (Table 4). However, none of the interaction tests between PAC and any of the stratified subgroups were statistically significant. Additionally, recognizing that medications may influence PAC levels and thereby indirectly affect our findings, we conducted a subgroup analysis based on the use of various drugs. The results across all subgroups were consistent with the overall findings. Consequently, this analysis strengthens our conclusion that elevated PAC increases the risk of PAD in hypertensive patients, and this association is not influenced by the use of these medications (Supplementary Table S6).

**Table 4:** Association between PAC and PAD in various subgroups.

Subgroups	*N*	OR (95% CI)	*P* for interaction
Gender			0.491
Female	5,398	1.08 (1.04, 1.11)	
Male	7,759	1.06 (1.04, 1.08)	
Age, years			0.616
<60	9,374	1.06 (1.04, 1.09)	
≥60	3,783	1.07 (1.05, 1.10)	
Current smoking			0.640
No	8,528	1.07 (1.05, 1.09)	
Yes	4,629	1.06 (1.04, 1.09)	
Current drinking			0.873
No	8,955	1.07 (1.05, 1.09)	
Yes	4,202	1.06 (1.03, 1.10)	
Hyperlipemia			0.542
No	6,281	1.07 (1.05, 1.10)	
Yes	6,876	1.06 (1.04, 1.09)	
Diabetes			0.320
No	10,933	1.07 (1.05, 1.09)	
Yes	2,224	1.05 (1.02, 1.09)	
CHD			0.473
No	12,054	1.07 (1.05, 1.09)	
Yes	1,103	1.05 (1.01, 1.10)	
BMI, kg/m^2^			0.175
<24	2,541	1.08 (1.04, 1.12)	
24–28	5,749	1.08 (1.05, 1.11)	
≥28	4,867	1.05 (1.02, 1.08)	
PRA, ng/ml/h			0.425
<1	3,127	1.06 (1.02, 1.09)	
≥1	10,030	1.07 (1.05, 1.09)	

Models were adjusted for all covariates other than the stratifcation variable.

BMI, body mass index; PRA, plasma renin activity; CHD, coronary heart disease.

## Discussion

In this cross-sectional investigation, a significant correlation between PAC and the prevalence of PAD was elucidated within a sample of hypertensive patients. Employing non-linear regression analysis, we discerned an inflection point at a PAC threshold of 17.00 ng/dl, delineating distinct patterns of association on either side of this threshold. PAC exhibited a positive correlation with PAD prevalence to the right of the inflection point, whereas no such correlation was observed to the left. The identification of this inflection point signifies a critical threshold above which the incidence of PAD appears to augment, implying that excessive aldosterone may exert a substantial impact on vascular integrity. These findings advocate for further elucidation of the intricate role of aldosterone in vascular pathophysiology and its clinical ramifications, particularly in the management of PAD associated with hypertension.

The relationship between antihypertensive therapies, aldosterone levels, and PAD outcomes is complex. A meta-analysis of 7 randomized controlled trials (RCTs) involving 71,971 patients revealed that calcium channel blockers (CCB) reduced the odds of PAD development in hypertensive patients compared to placebo or active treatment, with an OR of 0.70 (0.58, 0.86) (33). While these medications may offer protective effects against PAD, their impact on limb-specific outcomes remains inconsistent. For instance, Khan et al. found no significant reduction in major amputations with the use of angiotensin-converting enzyme inhibitors (ACEIs) or angiotensin receptor blockers (ARBs) (OR 1.04, 95% CI: 0.91, 1.18), whereas Elsayed et al. reported a modest improvement [hazard ratio (HR) 0.93, 95% CI: 0.87, 0.99] in a propensity-matched cohort (34, 35). This inconsistency may be attributed to varying rates of aldosterone “escape” across studies, a phenomenon where chronic inhibition of the renin-angiotensin-aldosterone system (RAAS) triggers compensatory aldosterone elevation through non-ACE pathways, potentially offsetting the vascular protective effects in PAD (36, 37). Furthermore, a review of studies involving 47,612 participants highlighted a significant association between diuretic use and increased amputation risk in PAD patients, with a meta-analysis showing an OR of 1.75 (1.53, 1.99) (38). To better understand the differential effects of drug classes, we conducted subgroup analyses stratified by ACEIs/ARBs, beta-blockers, calcium channel blockers (CCB), diuretics, lipid-lowering drugs, antidiabetic drugs, and antiplatelet drugs. The results remained consistent, reinforcing the stability of our findings. This study provides new insights into the interplay between antihypertensive drugs, PAC, and PAD outcomes.

The intricate relationship between PAC and PAD in hypertensive populations is not yet fully delineated. Aldosterone is posited to exert deleterious effects on the vasculature through various mechanisms, including the promotion of atherosclerosis, endothelial dysfunction, vascular inflammation, and vascular remodeling (39, 40). PAD is initiated by the pathological deposition of lipid and fibrous substances within the arterial walls of the lower limbs (41), with subclinical atherosclerosis identified as a significant risk factor for PAD (17). A prospective study by Marieke et al. (42) involving 2,758 patients with established coronary artery disease revealed that baseline aldosterone levels were positively correlated with the involvement of multiple vascular territories and the presence of carotid artery stenosis, and negatively associated with ABI. These findings suggest a link between aldosterone signaling and atherosclerotic processes. However, to date, no studies have directly assessed the association between PAC and PAD in hypertensive populations.

Aldosterone excess has been implicated in endothelial cell (EC) and vascular smooth muscle cell (VSMC) dysfunction (17, 18, 43). Current research supports the role of increased plasma aldosterone levels in the pathogenesis of impaired vascular relaxation and vascular stiffness. The intricate interplay between ECs and VSMCs is crucial for the modulation of vascular function and tone. ECs exert their vasodilatory effects on VSMCs principally through the production of nitric oxide (NO). NO production by ECs inhibits VSMC proliferation and migration, thereby preventing vascular stiffening (44, 45). Nishizaka et al. (46). demonstrated that elevated PAC is correlated with impaired flow-mediated vasodilation in hypertensive patients. NO, released by the endothelium, induces vasodilation, reduces vascular resistance, enhances regional blood flow, and lowers blood pressure (47). Conversely, high aldosterone concentrations can suppress NO synthesis and release (46, 48), potentially increasing EC stiffness and adversely affecting vascular function (49). Chronic exposure to elevated aldosterone levels may exacerbate endothelial dysfunction, contributing to the development of PAD (50).

Inflammation is another critical factor. Aldosterone overproduction can stimulate the local production of Ang II, a key mediator in the process involving transforming growth factor-beta 1 (TGF-β1) (16, 51). TGF-β1 and highly pro-inflammatory interleukins such as IL-1β and IL-18 can stimulate excessive extracellular matrix fibroblasts to differentiate into active myofibroblasts (52, 53). Myofibroblasts promote excessive fibrosis of the vascular wall, reducing vascular elasticity, and leading to thickening and hardening of the vascular wall (54, 55). It is noteworthy that the renin-angiotensin system (RAS) and TGF-β1 are not only key mediators of cardiac adaptations to hemodynamic overload but also critically involved in the pathogenesis of cardiac hypertrophy and failure. Ang II upregulates TGF-β1 expression via activation of the angiotensin type 1 (AT1) receptor in cardiac myocytes and fibroblasts, and induction of this cytokine is absolutely required for Ang II-induced cardiac hypertrophy *in vivo*. This interplay between Ang II and TGF-β1 may provide insight into the potential mechanisms by which aldosterone influences PAD pathogenesis. In conclusion, the multifaceted effects of aldosterone on the vasculature suggest a potential role in the development and progression of PAD. Further research is warranted to elucidate the direct relationship between PAC and PAD in hypertensive populations, which could have significant implications for the management and treatment of these conditions.

The present study is bolstered by its large sample size, which facilitates robust statistical analysis and enhances the precision of our estimates. The implementation of rigorous inclusion criteria has effectively minimized selection bias, ensuring a more accurate representation of the hypertensive population under study. Additionally, our research introduces a novel perspective by uncovering a nonlinear association between PAC and the prevalence of PAD, a finding that could significantly inform future investigative and clinical endeavors. Despite these strengths, our study is not without limitations that warrant consideration. Firstly, the cross-sectional design limits our ability to infer causality, a shortcoming inherent to this type of observational research. Longitudinal studies are needed to confirm the temporality and directionality of the observed associations. Secondly, the measurement of PAC at a single time point may not fully capture the dynamic fluctuations in aldosterone levels that occur over time, potentially leading to misestimation of the true exposure-risk relationship. Thirdly, while we adjusted for the use of antihypertensive medications (e.g., diuretics, ACEIs/ARBs, beta-blockers) in our analyses, detailed data on drug dosages, duration of use, and combination therapies were unavailable. This limitation introduces the possibility of residual confounding, as these factors may modulate aldosterone levels and obscure or exaggerate the observed associations. Fourthly, the exclusive use of ABI for PAD diagnosis, while practical, may not capture the full spectrum of PAD cases, particularly in the early stages of the disease. The incorporation of more sensitive diagnostic tools, such as computed tomography angiography (CTA), could enhance the accuracy of PAD detection (56–61). Lastly, the study's focus on a hypertensive patient population limits the generalizability of our findings to other demographic groups. Future research should aim to include diverse populations to ascertain the broader applicability of our observations regarding the relationship between PAC and PAD.

## Conclusions

In conclusion, our large cross-sectional analysis establishes a significant nonlinear association between PAC and the prevalence of PAD in hypertensive patients, with a critical threshold at 17.00 ng/dl. Above this threshold, each unit increase in PAC is associated with a 9% increase in PAD prevalence. These findings highlight the potential role of aldosterone in vascular pathology and warrant further investigation into its clinical implications for PAD management in hypertensive individuals.

## Data Availability

The raw data supporting the conclusions of this article will be made available by the authors, without undue reservation.
