# Supplementary material for: Association of plasma aldosterone concentration with peripheral artery disease in hypertensive patients: insights from a large cross-sectional analysis
Source: Front Cardiovasc Med. 2025 Mar 25;12:1549878. doi: 10.3389/fcvm.2025.1549878 (PMC11977419; doi:10.3389/fcvm.2025.1549878)
Supplement: Supplementary file 1 [file Presentation1.pdf]

Supplementary Material

Supplemental material and methods

Description of anthropometric measurements and data collection

Anthropometric data were collected by trained nursing staff adhering to a standardized protocol, with weight measured to the nearest 0.1 kg and height to 0.1 cm, ensuring precise documentation of these fundamental metrics. To control for variability, participants were asked to wear light clothing and remove headwear and footwear. Blood pressure was measured using a certified automated electronic sphygmomanometer (Omron Corporation) after a 10-minute period of quiet repose in a calm, well-lit setting, with the average of multiple readings used to determine systolic and diastolic blood pressure values. Body Mass Index (BMI) was calculated using the standard formula: weight in kilograms divided by the square of height in meters (kg/m^2).

Lifestyle factors, including alcohol consumption and smoking habits, were documented as binary variables, categorized as either never/former or current. Current smokers were defined as those who smoked at least one cigarette daily for a minimum of six months. Current alcohol consumption was identified in individuals who reported the weekly intake of at least one alcoholic beverage over the preceding month.

Fasting blood samples were collected in the morning after an overnight fast, with participants instructed to abstain from food intake. An extensive panel of laboratory parameters was assessed, including fasting plasma glucose (FPG), high-density lipoprotein cholesterol (HDL-C), low-density lipoprotein cholesterol (LDL-C), total cholesterol (TC), triglycerides (TG), serum uric acid (SUA), blood urea nitrogen (BUN), aspartate aminotransferase (AST), alanine aminotransferase (ALT), serum potassium (K^+), and homocysteine (Hcy). These parameters were quantified using an automated biochemical analyzer for accuracy and consistency. Serum creatinine (Scr) levels were determined using an enzymatic assay method.

To ensure accurate measurements of plasma aldosterone concentration (PAC) and plasma renin activity (PRA), hospitalized hypertensive individuals were either suspended from interfering drugs for 4-6 weeks or switched to slow-release verapamil and/or α 1-adrenergic antagonists. PRA was measured using an iodine angiotensin I (Ang I) radioimmunoassay kit (Northern Biotechnology Institutes, Beijing, China), known for its sensitivity and specificity. The Aldosterone/Renin Ratio (ARR) was calculated by dividing PAC by PRA, offering a valuable indicator of aldosterone production relative to renin activity [1].

Description of medical history assessment

Medical diagnoses were meticulously ascertained based on established clinical criteria. Hypertension was diagnosed if participants presented with a seated resting systolic

blood pressure (SBP) of ≥140 mmHg and/or diastolic blood pressure (DBP) of ≥90 mmHg, reported a history of hypertension, or were on antihypertensive medications at baseline assessment.

Diabetes mellitus was identified through fasting glucose levels ≥7.0 mmol/L, a 2-hour serum glucose ≥11.1 mmol/L following an oral glucose tolerance test, use of glucose-lowering medications, or self-reported history of diabetes.

Hyperlipidemia was characterized by total cholesterol levels ≥6.2 mmol/L, low-density lipoprotein cholesterol (LDL-C) levels ≥4.1 mmol/L, triglyceride levels ≥2.3 mmol/L, high-density lipoprotein cholesterol (HDL-C) levels below 1.0 mmol/L, use of lipid-lowering drugs, or a self-reported history of hyperlipidemia.

Coronary heart disease (CHD) was defined by a history of fatal or nonfatal myocardial infarction, unstable angina, or coronary revascularization procedures, including coronary artery bypass grafting or percutaneous transluminal coronary angioplasty. Myocardial infarction was diagnosed with elevated cardiac enzymes along with symptoms such as ischemic chest pain, evolving ST-T segment changes on an electrocardiogram (ECG), or new Q waves. Unstable angina was diagnosed based on hospitalization or outpatient follow-up, conforming to Braunwald's criteria, which encompass symptoms at rest or with minimal exertion lasting over 20 minutes (unless relieved by nitroglycerine), newly onset severe angina within the last month, or a crescendo pattern of chest discomfort more severe, prolonged, or frequent than previously experienced, without ST-segment elevation myocardial infarction signs on ECGs, and/or positive cardiac biomarkers.

Primary aldosteronism (PA) was suspected in patients with an aldosterone-renin ratio (ARR) of at least 20 ng/dl per ng/ml per hour and a plasma aldosterone concentration (PAC) of at least 12 ng/dl [2].

Details of the statistical analyses

To address the challenge of missing data within our study, we employed a nonparametric imputation method known as missForest, which is implemented in the R programming environment. This approach leverages the power of random forest algorithms to predict missing values for each variable with missing data points. By utilizing the information from the remaining variables within the dataset, the missForest procedure effectively estimates and substitutes the missing values, thereby preserving the integrity of our dataset for subsequent analyses.

Baseline characteristics were analyzed using descriptive statistics. Participants were categorized into tertiles based on plasma aldosterone concentration (PAC) levels, which served as the foundational stratification for our analysis. Categorical variables were described as frequency and percentage. Continuous variables were described as mean (± standard deviation [SD]) for normally distributed data and as geometric mean and 95% confidential interval (CI) for data not normally distributed. Continuous variables were compared using one-way ANOVA, while categorical variables were

compared using the chi-square test.

We rigorously assessed model assumptions by evaluating multicollinearity using variance inflation factors (VIFs), with a threshold of VIF > 5 indicating potential multicollinearity (Table S1). Univariate logistic regression analysis was performed to assess the impact of the clinical and biochemical indicators on the risk of PAD (Table S2). This step was critical in ensuring the robustness of our multivariate logistic regression models, which were employed to estimate odds ratios (ORs) and 95% confidence intervals (CIs) for the relationship between PAC and PAD prevalence. Four models were constructed: the crude model was not adjusted; model 1 was adjusted for age, sex, smoking, drinking, diabetes, CHD, hyperlipidemia, BMI, SBP, and DBP; model 2 was additionally adjusted for FPG, HDL-C, LDL-C, TC, TG, UA, BUN, Scr, AST/ALT, K^+, and Hcy; model 3 was additionally adjusted for ACEIs/ARBs, beta blockers, calcium channel blockers, diuretics, lipid-lowering drugs, antidiabetic drugs, and antiplatelet drugs.

To elucidate the dose-response relationship between PAC and PAD prevalence, a generalized additive model was conducted. This method allowed for a flexible and non-parametric assessment of the relationship, unmasking potential non-linear trends. A recursive algorithm was adeptly used to calculate inflection points, which were pivotal in constructing threshold analyses. These analyses, employing a two-segment binary logistic model, dissected the relationship between PAC and PAD prevalence at different levels of PAC. Stratified analyses were performed according to age, sex , smoking status, alcohol drinking status, obesity status, comorbidities (diabetes, CHD, hyperlipemia), and PRA. P values for interactions were evaluated using interaction terms and likelihood ratio tests.

Multiple sensitivity analyses were presented here to examine the robustness of our findings. These included the exclusion of individuals suspected of having PA to preclude the confounding influence of this endocrine disorder on our analyses. Additionally, to address the potential impact of extreme values on our regression models, we excluded data points corresponding to PAC that fell outside the 1st and 99th percentiles. This approach is recognized for its efficacy in reducing the skewing effects of outliers. Furthermore, to maintain the analytical rigor, participants with incomplete covariate data were also excluded from the dataset. This exclusionary criterion is instrumental in preserving the reliability of our multivariate analyses.

All statistical analyses were conducted using R software, version 4.1.1 (R Core Team, Vienna, Austria). The statistical tests were two-tailed, with a p-value of less than 0.05 deemed to indicate statistical significance.

Supplementary Tables

Table S1. Collinearity diagnostics steps.

Variables	Step 1	Step 2
PAC	1.2	1.2
Sex	2.8	2.8
Age	1.5	1.5
Current smoking	1.8	1.8
Current drinking	1.6	1.6
Diabetes	2.6	2.6
CHD	1.2	1.2
Hyperlipidemia	1.5	1.5
Body mass index	1.2	1.2
SBP	2.0	2.0
DBP	2.3	2.3
FPG	1.9	1.9
HDL-C	2.8	1.4
LDL-C	8.8	1.4
TC	12.1	NA
TG	4.1	1.5
SUA	1.7	1.7
BUN	1.3	1.3
Scr	2.0	2.0
AST/ALT	1.3	1.3
Hcy	1.2	1.2
ACEIs/ARBs	1.2	1.2
Beta-blockers	1.1	1.1
Calcium channel blockers	1.1	1.1
Diuretics	1.2	1.2
Lipid-lowering drugs	2.0	2.0
Antidiabetic drugs	1.8	1.8
Antiplatelet drugs	1.9	1.9
K^+	1.0	1.0
PRA	1.2	1.2
ARR	1.2	1.2

VIF= 1/(1-R2). VIF step-by-step screening method: Calculate the VIF of each variable. If the maximum VIF value is ⩾5, remove the variable with the maximum VIF value.

VIF: variance inflation factors. For other abbreviations, see Table 1.

Table S2. Association of PAD with the clinical and biochemical indicators.

Variables	OR (95% CI)	P value
Gender	1.01 (0.76, 1.33)	0.967
Age	1.00 (0.99, 1.02)	0.667
Current smoking	1.20 (0.90, 1.60)	0.204
Current drinking	2.78 (2.10, 3.68)	<0.001
Diabetes	1.45 (1.04, 2.02)	0.028
CHD	1.92 (1.30, 2.84)	0.001
Hyperlipemia	1.13 (0.85, 1.49)	0.043
BMI	1.07 (1.03, 1.11)	<0.001
SBP	1.00 (1.00, 1.01)	0.438
DBP	0.99 (0.98, 1.00)	0.093
FPG	1.15 (1.01, 1.30)	0.034
HDL-C	0.41 (0.24, 0.71)	0.001
LDL-C	1.15 (0.99, 1.34)	0.077
TC	1.06 (0.93, 1.21)	0.379
TG	1.11 (0.99, 1.25)	0.065
SUA	1.01 (1.00, 1.01)	<0.001
BUN	1.12 (1.01, 1.23)	0.024
Scr	1.02 (1.01, 1.02)	<0.001
AST/ALT	0.76 (0.48, 1.20)	0.242
Hcy	1.04 (1.01, 1.07)	0.003
ACEIs/ARBs	0.51 (0.38, 0.69)	<0.001
K^+	1.14 (0.75, 1.72)	0.542
Beta-blockers	0.60 (0.40, 0.91)	0.015
Calcium channel blockers	0.47 (0.35, 0.62)	<0.001
Diuretics	1.27 (0.92, 1.75)	0.145
Lipid-lowering drugs	0.47 (0.29, 0.72)	0.001
Antidiabetic drugs	0.47 (0.24, 0.91)	0.026
Antiplatelet drugs	0.31 (1.22, 0.86)	0.011

Abbreviations: BMI, body mass index; SBP, systolic blood pressure; DBP, diastolic blood pressure; FPG: fasting plasma glucose; HDL-C, high-density lipoprotein cholesterol; LDL-C, low-density lipoprotein cholesterol; TC, total cholesterol; TG, total triglyceride; SUA, serum uric acid; BUN, blood urea nitrogen; Scr, serum creatinine; AST, aspartate aminotransferase; ALT, alanine aminotransferase; Hcy, homocysteine; K^+, serum potassium; PAD, peripheral artery disease.

Table S3. Association between PAC and the prevalence of PAD after excluding individuals that were suspected of having PA.

Exposure	Crude Model		Model 1		Model 2		Model 3	
	OR (95% CI)	P value	OR (95% CI)	P value	OR (95% CI)	P value	OR (95% CI)	P value
PAC(Per unit increase)	1.07 (1.05, 1.08)	<0.001	1.07 (1.05, 1.08)	<0.001	1.07 (1.05, 1.09)	<0.001	1.06 (1.04, 1.08)	<0.001
Tertiles of PAC								
Tertile 1	Reference		Reference		Reference		Reference	
Tertile 2	0.94 (0.62, 1.41)	0.752	0.95 (0.63, 1.43)	0.797	0.96 (0.63, 1.44)	0.829	0.96 (0.64, 1.46)	0.862
Tertile 3	1.91 (1.34, 2.72)	0.001	1.95 (1.37, 2.78)	0.001	1.89 (1.32, 2.72)	0.001	1.81 (1.25, 2.62)	0.001
P for trend	1.44 (1.19, 1.73)	0.001	1.45 (1.20, 1.74)	<0.001	1.42 (1.18, 1.72)	0.001	1.39 (1.15, 1.68)	0.010

Crude Model: Unadjusted.

Model 1: Adjust for age, sex, current smoking, current drinking, diabetes, CHD, hyperlipidemia, BMI, SBP, DBP.

Model 2: Model 1 plus adjustment for FPG, HDL-C, LDL-C, TC, TG, SUA, BUN, Scr, AST/ALT, Hcy, K^+.

Model 3: Model 2 plus adjustment for ACEIs/ARBs, beta blockers, calcium channel blockers, diuretics, lipid-lowering drugs, antidiabetic drugs, antiplatelet drugs.

Abbreviations: PAC, plasma aldosterone concentration; PAD, peripheral artery disease; PA, primary aldosteronism; OR, odds ratio; CI, confidence interval.

Table S4. Association between PAC and the prevalence of PAD after excluding participants with extreme PAC values.

Exposure	Crude Model		Model 1		Model 2		Model 3	
	OR (95% CI)	P value	OR (95% CI)	P value	OR (95% CI)	P value	OR (95% CI)	P value
PAC(Per unit increase)	1.07 (1.05, 1.09)	<0.001	1.07 (1.05, 1.09)	<0.001	1.07 (1.05, 1.09)	<0.001	1.06 (1.04, 1.08)	<0.001
Tertiles of PAC								
Tertile 1	Reference		Reference		Reference		Reference	
Tertile 2	0.97 (0.65, 1.45)	0.875	0.98 (0.65, 1.46)	0.910	0.97 (0.64, 1.45)	0.872	0.97 (0.65, 1.46)	0.897
Tertile 3	1.95 (1.38, 2.77)	0.001	1.98 (1.39, 2.81)	0.001	1.83 (1.27, 2.62)	0.001	1.75 (1.21, 2.52)	0.003
P for trend	1.45 (1.21, 1.74)	<0.001	1.46 (1.21, 1.75)	<0.001	1.40 (1.16, 1.68)	0.001	1.36 (1.13, 1.64)	0.001

Crude Model: Unadjusted.

Model 1: Adjust for age, sex, current smoking, current drinking, diabetes, CHD, hyperlipidemia, BMI, SBP, DBP.

Model 2: Model 1 plus adjustment for FPG, HDL-C, LDL-C, TC, TG, SUA, BUN, Scr, AST/ALT, Hcy, K^+.

Model 3: Model 2 plus adjustment for ACEIs/ARBs, beta blockers, calcium channel blockers, diuretics, lipid-lowering drugs, antidiabetic drugs, antiplatelet drugs.

Abbreviations: PAC, plasma aldosterone concentration; PAD, peripheral artery disease; OR, odds ratio; CI, confidence interval.

Table S5. Association between PAC and the prevalence of PAD after excluding participants with missing covariate data.

Exposure	Crude Model		Model 1		Model 2		Model 3	
	OR (95% CI)	P value	OR (95% CI)	P value	OR (95% CI)	P value	OR (95% CI)	P value
PAC(Per unit increase)	1.07 (1.05, 1.09)	<0.001	1.07 (1.05, 1.09)	<0.001	1.07 (1.05, 1.09)	<0.001	1.06 (1.04, 1.08)	<0.001
Tertiles of PAC								
Tertile 1	Reference		Reference		Reference		Reference	
Tertile 2	0.88 (0.58, 1.34)	0.552	0.87 (0.57, 1.34)	0.535	0.86 (0.56, 1.32)	0.501	0.86 (0.56, 1.33)	0.505
Tertile 3	1.87 (1.30, 2.68)	0.001	1.85 (1.28, 2.65)	0.001	1.74 (1.20, 2.52)	0.004	1.64 (1.12, 2.39)	0.010
P for trend	1.43 (1.18, 1.72)	0.001	1.42 (1.17, 1.72)	0.001	1.37 (1.13, 1.67)	0.001	1.33 (1.09, 1.61)	0.001

Crude Model: Unadjusted.

Model 1: Adjust for age, sex, current smoking, current drinking, diabetes, CHD, hyperlipidemia, BMI, SBP, DBP.

Model 2: Model 1 plus adjustment for FPG, HDL-C, LDL-C, TC, TG, SUA, BUN, Scr, AST/ALT, Hcy, K^+.

Model 3: Model 2 plus adjustment for ACEIs/ARBs, beta blockers, calcium channel blockers, diuretics, lipid-lowering drugs, antidiabetic drugs, antiplatelet drugs.

Abbreviations: PAC, plasma aldosterone concentration; PAD, peripheral artery disease; OR, odds ratio; CI, confidence interval.

Table S6 Association between PAC and PAD in various subgroups.

Subgroups	N	OR (95% CI)	P for interaction
ACEIs/ARBs			0.014
No	6625	1.09 (1.07, 1.11)	
Yes	6532	1.05 (1.02, 1.07)	
Beta-blockers			0.606
No	10466	1.07 (1.05, 1.09)	
Yes	2691	1.06 (1.03, 1.09)	
Calcium channel blockers			0.486
No	4603	1.08 (1.04, 1.11)	
Yes	8554	1.06 (1.04, 1.08)	
Diuretics			0.626
No	10441	1.07 (1.04, 1.09)	
Yes	2716	1.06 (1.03, 1.08)	
Lipid-lowering drugs			0.701
No	10652	1.07 (1.05, 1.09)	
Yes	2505	1.06 (1.03, 1.09)	
Antidiabetic drugs			0.347
No	11962	1.07 (1.05, 1.09)	
Yes	1195	1.05 (0.99, 1.09)	
Antiplatelet drugs			0.268
No	11258	1.06 (1.04, 1.08)	
Yes	1899	1.08 (1.05, 1.11)	

Abbreviations: ACEIs, angiotensin-converting enzyme inhibitors; ARBs, angiotensin receptor blockers.

References

[1] Ng E, Gwini SM, Libianto R, et al. Aldosterone, Renin, and Aldosterone-to-Renin Ratio Variability in Screening for Primary Aldosteronism. J Clin Endocrinol Metab. 2022. 108(1): 33-41.

[2] Lin M, Heizhati M, Gan L, et al. Higher aldosterone is associated with increased renal impairment risk in patients with hypertension and abnormal glucose metabolism: a longitudinal study. J Hypertens. 2022. 40(3): 561-569.
